# Identification of Novel Genetic Alterations in Samples of Malignant Glioma Patients

**DOI:** 10.1371/journal.pone.0082108

**Published:** 2013-12-16

**Authors:** Vedrana Milinkovic, Jasna Bankovic, Miodrag Rakic, Tijana Stankovic, Milica Skender-Gazibara, Sabera Ruzdijic, Nikola Tanic

**Affiliations:** 1 University of Belgrade, Institute for Biological Research “Sinisa Stankovic”, Department of Neurobiology, Belgrade, Republic of Serbia; 2 Clinical Center of Serbia, Clinic for Neurosurgery, Belgrade, Republic of Serbia; 3 University of Belgrade, School of Medicine, Institute of Pathology, Belgrade, Republic of Serbia; University Hospital of Navarra, Spain

## Abstract

Glioblastoma is the most frequent and malignant human brain tumor. High level of genomic instability detected in glioma cells implies that numerous genetic alterations accumulate during glioma pathogenesis. We investigated alterations in AP-PCR DNA profiles of 30 glioma patients, and detected specific changes in 11 genes not previously associated with this disease: *LHFPL3*, *SGCG*, *HTR4*, *ITGB1*, *CPS1*, *PROS1*, *GP2*, *KCNG2*, *PDE4D*, *KIR3DL3*, and *INPP5A*. Further correlations revealed that 8 genes might play important role in pathogenesis of glial tumors, while changes in *GP2*, *KCNG2* and *KIR3DL3* should be considered as passenger mutations, consequence of high level of genomic instability. Identified genes have a significant role in signal transduction or cell adhesion, which are important processes for cancer development and progression. According to our results, *LHFPL3* might be characteristic of primary glioblastoma, *SGCG*, *HTR4*, *ITGB1*, *CPS1*, *PROS1* and *INPP5A* were detected predominantly in anaplastic astrocytoma, suggesting their role in progression of secondary glioblastoma, while alterations of *PDE4D* seem to have important role in development of both glioblastoma subtypes. Some of the identified genes showed significant association with *p53*, *p16*, and *EGFR*, but there was no significant correlation between loss of *PTEN* and any of identified genes. In conclusion our study revealed genetic alterations that were not previously associated with glioma pathogenesis and could be potentially used as molecular markers of different glioblastoma subtypes.

## Introduction

Cancer is a genetic disease characterized by DNA sequence changes, copy number aberrations, chromosomal rearrangements and modification in DNA methylation leading to compromised regulatory mechanisms governing cell proliferation and homeostasis. Studies carried out over the past three decades suggest that malignant gliomas, like other cancers, represent a consequence of the accumulation of genetic alterations, although their nature and exact number required for tumorigenesis remain unclear.

Glioblastoma is the most frequent and aggressive brain tumor. Majority of glioblastomas (GBM, WHO grade IV) develop *de novo* (primary glioblastomas) without clinical or histological evidence of a less malignant precursor lesion, while others, progressing from low-grade diffuse astrocytoma or anaplastic astrocytoma (AA), represent rare secondary glioblastomas. Despite a similar histological appearance, primary and secondary glioblastomas are distinct tumor entities that originate from different precursor cells containing different genetic alterations [1]. Epidermal growth factor receptor (*EGFR*) amplification and *PTEN* mutations are genetic alterations typical of primary glioblastomas, whereas *p53* mutations are early and frequent genetic alterations in the pathway leading to secondary glioblastomas. Furthermore, mutations in the active site of isocitrate dehydrogenase 1 (*IDH1*) were associated with secondary GBMs [2]. On the other hand, LOH 10q and alterations of p16^INK4a^/RB1 pathway seem to be important in the development of both primary and secondary glioblastomas [3]. Besides these frequently altered pathways, there is evidence of large number of genetic alterations in glioblastoma samples, reported in The Cancer Genome Atlas (TCGA) database. TCGA project enabled the integrated analyses of multi-dimensional genomic data collected from different platforms with the aim to better characterize and understand tumor origin, behavior and treatment [4].

However, further analyses are needed to identify additional potentially useful genetic alterations for the classification and targeted therapy of GBMs. Besides, significant level of genomic instability and heterogeneity detected in glial tumors confirm that they evolve along a multitude of pathways rather than along a single defined pathway [5], [6], [7]. Even though only a few specific genetic pathways are consistently highlighted, there are undoubtedly complex interactions among them as well as with additional yet unknown factors. Reliable molecular markers are needed to enable the identification of patients at risk for developing GBM, improve the early detection and appropriate diagnosis, as well as to provide molecular profile for better prediction of patient outcome and response to therapy.

A number of techniques based on PCR, hybridization, and conformation changes, as well as modern high-throughput genome wide techniques can be employed for the detection of specific mutations in cancer cells. AP-PCR DNA fingerprinting method has numerous advantages over conventional methods, mainly because no prior knowledge of the genome under investigation is required and because it allows the screening of the whole genome including non coding DNA regions. Furthermore, AP-PCR is a reliable, inexpensive method that does not require complex equipment, does providing highly reproducible patterns of amplified fragments which faithfully reflect differences in DNA sequences and/or relative abundance of the templates and enable detection of genetic alterations in tumor tissue [8]. Finally, the possibility of further analysis of variant bands by reamplification, cloning, and sequencing enables rapid identification of genes probably linked to the development and progression of malignant tumor [9]. AP-PCR has already proven to be highly informative for analysis of cancer associated somatic mutations since it has been implemented in the analyzes of various cancers, including pancreatic and colorectal carcinomas [10], [11], as well as lung [12], [13], and breast cancers [14].

We applied AP-PCR for the detection of anonymous multiple genetic alterations in 30 patients with AA and GBM. Following our previous study of genomic instability in glioma patients [15] we analyzed altered sequences in tumor DNA profiles with the aim to identify genes specific for glioma pathogenesis. Furthermore, we examined identified genes in relation to genomic instability, clinicopathological parameters, and patients' survival. Also, we tested the association of the most frequently present genetic alterations in primary and secondary glioblastomas with newly identified genes with the aim to recognize potential molecular markers of different glioblastoma subtypes.

## Materials and Methods

### Ethics Statement

The samples were collected and used after obtaining informed consents and approval from the Ethical Committee of Clinical Center of Serbia (approval number 3672/1), in accordance with the ethical standards laid down in the 1964 Declaration of Helsinki, Laws of Republic of Serbia, as well as GCP guidelines. All participants provided their written informed consent to participate in this study. The form of informed consent was approved by the Ethical Committee of Clinical Center of Serbia.

### Tissue samples

Paired cancer tissue and blood samples were collected from 30 patients who underwent surgery at Neurosurgery Clinic, Clinical Center of Serbia. The specimens were frozen in liquid nitrogen, where they were kept until DNA extraction. All patients had a histologically confirmed diagnosis of anaplastic astrocytoma (WHO grade III; n = 8) or glioblastoma multiforme (WHO grade IV; n = 22) according to the 2007 WHO classification [16]. All grade IV tumors were considered primary (*de novo*) because the glioblastoma diagnosis was made at the first biopsy, without clinical or histopathological evidence of a less malignant precursor lesion. The 30 patients included 19 men and 11 women, with a median age of 56.9 years (within the range of 20 to 84 years). The median overall survival was 11 months. Patients received neither radio- nor chemotherapy before surgery.

### Immunohistochemical analysis

Following routine hematoxylin-eosin method for staining of paraffin tissue sections [15], we performed immunohistochemical staining for p53 to further characterize our samples. Sections of tissue were subsequently heated in phosphate buffered saline (PBS), and stained with the streptavidin-biotin technique using antibody against p53 protein (Dako, Monoclonal Rabbit Anti-Human Antibody, dilution 1∶50), according to the manufacturer's instructions.

### DNA extraction

DNA was extracted using the phenol/chloroform/isoamyl alcohol method [17]. The quality of the DNA was verified by electrophoresis on 0.8% agarose gel. The DNA concentration was assessed spectrophotometrically.

### AP-PCR DNA fingerprinting

AP-PCR DNA fingerprinting, was used to compare DNA profiles of paired tumor and blood samples of the same patient [15]. In short, seven primers were tested for the ability to generate informative fingerprints that distinguish tumor from normal tissue. Optimization of AP-PCR reactions was done for each primer according to Cobb [18] and included the search for conditions that would yield profiles of moderate complexity in order to simplify the analysis [19]. Primer sequences, reaction conditions, and amplification profiles were described previously [15].

AP-PCR products were separated on 6–8% non-denaturing polyacrylamide (PAA) gels and visualized by silver staining. Gel images were acquired with the Multi-Analyst/PC Software Image Analysis System (Bio Rad Gel Doc 1000). Digitized images were loaded into the specialized public software Image J (National Institute of Health, USA, www.rsb.info.nih.gov/ij) and analyzed by the image enhancement function ‘adapthisteq’ as previously described [15].

### Isolation, cloning and sequencing of variant electrophoretic bands

Twenty selected DNA bands with altered mobility were further characterized. The PCR amplicons resolved on the silver stained PAA gels were gently removed with a hypodermic 22-gauge needle pre-wetted with the PCR master mix solution. The needle was dipped in the PCR master mix for 2 min and then discarded. The PCR products were reamplified with the same primers used for AP-PCR reactions at high-stringency conditions specific for each particular primer [20]. The reamplified material was administrated on 1.5% agarose gels, purified using DNA Extraction Kit (Fermentas Life Sciences, Lithuania) and cloned with GeneJet™ PCR Cloning Kit (Fermentas Life Sciences, Lithuania) according to manufacturer's instructions. Plasmids were purified using GeneJet™ Plasmid Miniprep Kit (Fermentas Life Sciences, Lithuania). Sequences were determined on ABI Prism 3130 Genetic Analyzer automated sequencer (Applied Biosystems, Foster City, CA, USA) using BigDye Terminator v3.1 Cycle Sequencing Kit (Applied Biosystems, Foster City, CA, USA). Sequencing was performed in both directions on several clones for each selected DNA band. The obtained sequences were analyzed and identified using BLAST software in the NCBI GenBank and EBI (Sanger Institute) database.

### Analysis of alterations in *p53*, *PTEN*, and *p16* tumor suppressor genes

Alterations of *p53* tumor suppressor gene were analyzed in our previous study [15]. Frequently mutated exons of *p53* gene (5–9) were amplified and screened for mutations by PCR-SSCP (Single Strand Conformation Polymorphism) analysis according to Orita et al. [21]. Detected mutations were confirmed by sequencing with Applied Biosystems Incorporated dye terminator sequencing kit according to the manufacturer's specifications on an ABI Prism 3130 automated sequencer (Applied Biosystems, Foster City, USA).

Tumor and blood DNA obtained from all 30 patients was used to study the loss of heterozygosity (LOH) of *p53*, *PTEN* and *p16* tumor suppressors. As described previously, LOH analysis of *p53* was performed by fragment analysis with highly polymorphic microsatellite markers (TP53pentanucleotide, TP53dinucleotide, D17S1537 and D17S786) specific for chromosomal region spanning *p53* gene locus [15]. Five polymorphic microsatellite markers lying within or flanking *PTEN* gene (D10S579, D10S1765, D10S215, AFM086wg9, and D10S541) were selected to cover deletions at the whole *PTEN* locus on chromosome 10q23. All forward primers were 5′-labeled with Fam, Vic, Ned, Pet, and Fam fluorescent dyes, respectively. The choice of the microsatellite markers and locus-specific PCR conditions was determined from published sources [22], [23].

Another set of 3 polymorphic microsatellite markers spanning the *INK4a/ARF* locus (D9S171, D9S1748 and D9S162) were selected to cover deletions on chromosome 9p21–23 [24]. All forward primers were 5′-labeled with Ned, Pet and Vic fluorescent dyes, respectively. PCR products for all LOH analyses were separated by capillary electrophoresis on an ABI Prism 3130 automated sequencer and sized using GeneScan −500 LIZ size standard (Applied Biosystems, Foster City, USA). The obtained data were analyzed with the GeneMapper software (Applied Biosystems). DNA extracted from peripheral blood of each patient was used as a reference. A marker was defined as noninformative (homozygote) when only 1 allelic peak was detected in the reference sample. Contrary to this, a marker was considered informative (heterozygote) when two major allelic peaks occurred in a blood specimen. The LOH score was calculated automatically by GeneMapper software according to the following equation: (peak height of normal allele 2)/(peak height of normal allele 1) divided by (peak height of tumor allele 2)/(peak height of tumor allele 1). A sample was considered to be a LOH candidate for particular locus if the ratio values were less than 0.67 and higher than 1.35. *p16* was also tested for the presence of homozygous deletions by a differential PCR method, according to conditions determined from published sources [12]. Briefly, a 199-bp fragment of INK4a/ARF locus from exon 2 was co-amplified with a 131-bp fragment of the adenine phosphoribosyltransferase (APRT) gene, which was used as internal control. Primer sequences were previously described by Hayashi et al. [25]. Forward primers were 5′-labeled with Fam fluorescent dye. Fluorescent PCR products were separated by capillary electrophoresis on an ABI Prism 3130 automated sequencer and sized using GeneScan −500 LIZ size standard (Applied Biosystems, Foster City, CA, USA). Obtained data were analyzed by fragment analysis with the GeneMapper software (Applied Biosystems, Foster City, CA, USA). The presence of homozygous deletions was determined according to ratio of peak intensities of *INK4a/ARF* and *APRT* in tumor samples relative to the same ratio in normal samples. A sample had homozygous deletion of *INK4a/ARF* locus and consequently *p16* tumor suppressor gene if the value of this proportion was higher than 2.

### Amplification status of *EGFR* gene

For differential PCR analysis, a 110-bp fragment of the *EGFR* gene on chromosome 7 was co-amplified with a 168-bp fragment of *β-actin (ACTB)* gene on the same chromosome. The primer sequences were as follows: 5′-AGC CAT GCC CGC ATT AGC TC-3′ (sense) and 5′-AAA GGA ATG CAA CTT CCC AA-3′ (antisense) for *EGFR*, and 5′-CTC TTT TCT TTC CCG ATA GGT-3′ (sense) and 5′-CTG GGA TGC TCT TCG ACC TC-3′ (antisense) for the *ACTB*. Genomic DNA (100 ng) was amplified with 0.8 µM of each primer, 1× KCl Buffer, 1.5 mM of MgCl_2_, 0.25 mM of dNTPs, and 1 U Taq polymerase (Fermentas, Thermo Scientific) in reaction volume of 25 µl. The PCR reaction was performed on the GeneAmp® PCR System 9700 (Applied Bioscience) under the following conditions: initial denaturation at 95°C for 10 minutes was followed by 30 cycles at 95°C for 1 minute, at 58°C for 1 minute and at 72°C for 1 minute, with final elongation at 72°C for 10 minutes. The PCR products were loaded on 2% agarose gels and stained with ethidium bromide. Multi-Analyst/PC Software Image Analysis System (Bio-Rad GelDoc 1000) was employed for densitometric analysis.

Ratio of the *EGFR/ACTB* score from tumor and blood tissue of each patient was calculated and values higher than 2 indicated presence of *EGFR* gene amplification.

### Statistical analysis

Significant differences between the data sets were determined by STATISTICA 6.0 software (StatSoft, Inc., Tulsa, USA). The correlations between identified genes and genomic instability, clinicopathological parameters and alterations of *p53*, *PTEN*, *p16*, and *EGFR* were evaluated using Fisher exact test. Survival analyses were performed using Kaplan and Meier product-limit method. The log rank test was used to assess the significance of the difference between pairs of survival probabilities. Overall survival was calculated from the day after surgery to the last follow-up examination or death of the patient. Statistical differences were considered significant when p was ≤0.05 (*), p≤0.01 (**) and p≤0.005 (***).

## Results

### Histopathological classification of tumor samples

**Figure 1:**
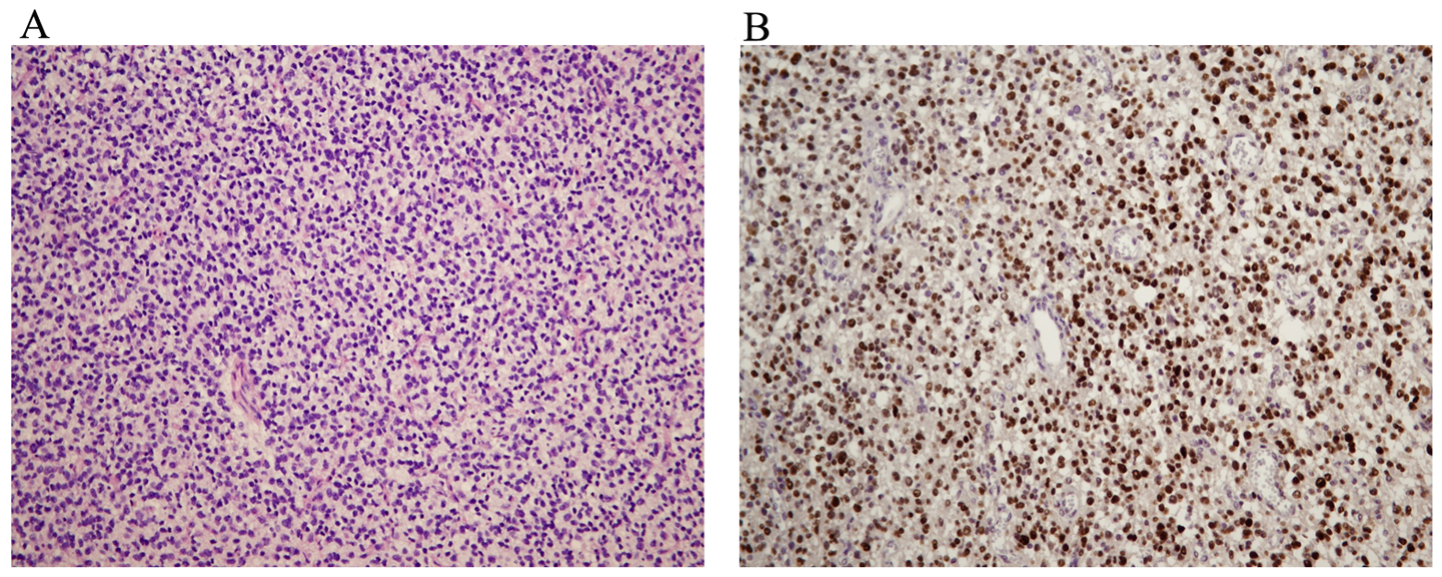
Immunohistochemical characterization of samples. Example of anaplastic astrocytoma (WHO grade III). (**A**) Cellular tumor with increased mitotic activity (HE ×200) (**B**) Diffuse nuclear p53 positivity of tumor cells (immunostaining ×250).

After staining of surgically removed tissues by routine hematoxylin-eosin method and immunohistochemical testing for p53 positivity (Fig. 1) we confirmed diagnosis of 8 anaplastic astrocytoma (WHO grade III) and 22 glioblastoma multiforme samples (WHO grade IV) according to WHO criteria (2007).

### Analysis of variant DNA fragments

Seven AP-PCR primers were used to discriminate normal from tumor tissue. Four of them produced informative AP-PCR DNA profiles containing explicit and countable differences between tumor and blood in all 30 patients that were analyzed. Observed differences were further classified as qualitative (mobility shifts in the banding pattern due to mutations at the primer-template interaction sites) and quantitative (altered band intensities representing amplifications or deletions of existing chromosomal material) and were used as a measurement of the level of total, microsatellite and chromosomal instability, as described in our previous paper [15]. Significant level of genomic instability was present in all samples, and based on the distribution of the frequency of DNA alterations, patients were assorted into two groups, with low or high level of genomic instability (Table 1).

**Table 1:** Distribution of samples according to the level of genomic instability.

Level of genomic instability	Number of patients
	30
**Genomic instability total**	
low≤0,3	11
high>0,3	19
**Microsatellite instability**	
low≤0,15	14
high>0,15	16
**Chromosomal instability**	
low≤0,16	13
high>0,16	17

**Figure 2:**
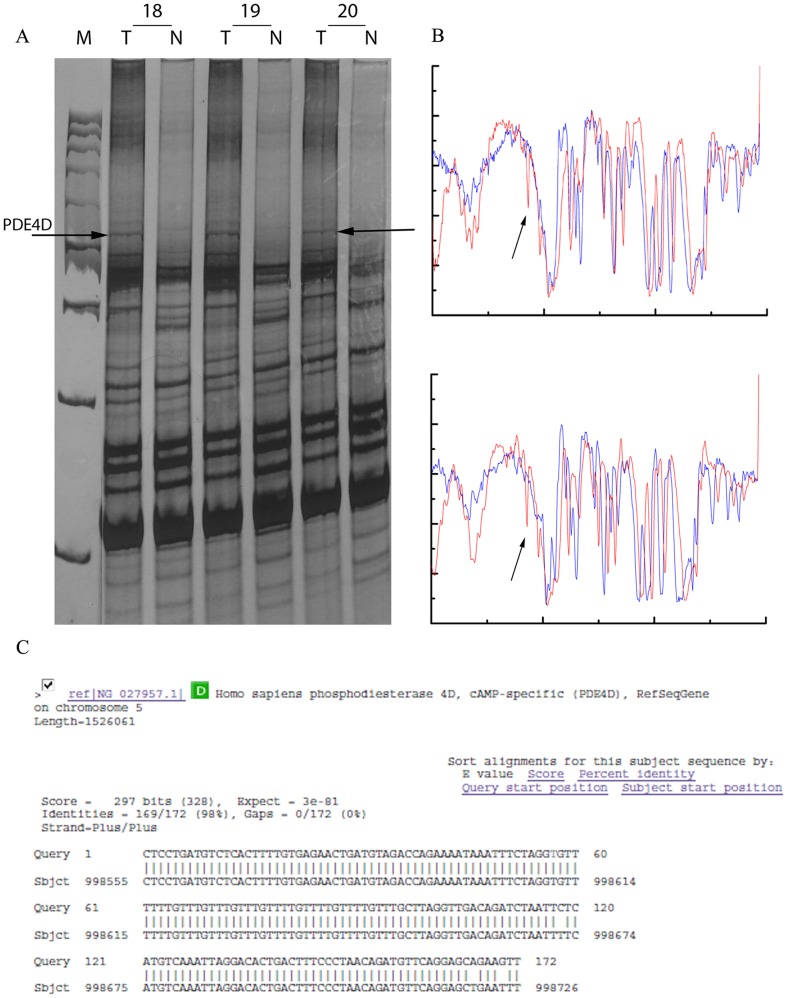
AP-PCR fingerprinting analysis of genomic instability in glioma samples. (**A**) AP-PCR profiles of tumor (T) and normal blood (N) tissues from glioma patients obtained using MDR antisense primer, separated on 6% non-denaturing polyacrylamide (PAA) gel; 18–20 represent patients; M –DNA ladder; arrows indicate altered electrophoretic bands in tumor DNA profiles that were excised, cloned, sequenced and identified as PDE4D. (**B**) contrast-limited adaptive histograms obtained using image enhancement function ‘adapthisteq’ of the specialized public software Image J, arrows indicate alterations; (**C**) the identity of the altered AP-PCR bands determined by BLAST homology search in the NCBI genBank and EBI Sanger databases.

The next step in our work was to identify some of the aberrant bands in DNA AP-PCR profiles common to more than 5 patients. Hence, twenty aberrant bands were retrieved from the PAA gels and cloned (Fig. 2A). Bands (amplicons) with the same electrophoretic mobility were isolated and characterized from at least two patients in order to confirm that they represent the same DNA sequence (Fig. 2B). Two clones of each band were sequenced. Obtained sequences were submitted for homology or identity search in NCBI GenBank and EBI (Sanger Institute) databases. Some of the sequences matched known genes and were easily identified, while others represented parts of certain contigs mapped on chromosomes 1, 2, 8q21–q23, 9, and 12, and their identification is yet to be determined (Fig. 2C).

**Figure 3:**
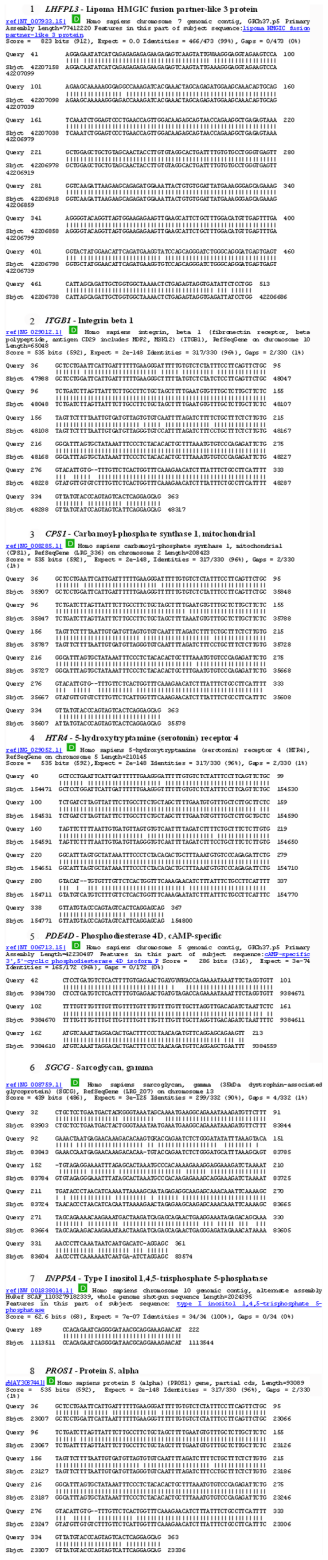
Identification of genetic alterations in tumor DNA fingerprints. The identity of the AP-PCR bands was determined by BLAST homology search in the NCBI GenBank and EBI Sanger databases. For each gene identified, numbers indicating beginning and the end of the region of homology in the GenBank sequence, overall sequence identity of the clone (%) and number of gaps are represented.

The following 11 genes were identified: lipoma HMGIC fusion partner-like 3 (*LHFPL3*); sarcoglycan, gamma (*SGCG*); 5-hydroxytryptamine (serotonin) receptor 4 (*HTR4*); integrin beta 1 (*ITGB1*); mitochondrial carbamoyl-phosphate synthetase 1 (*CPS1*); protein S (alpha) (*PROS1*); glycoprotein 2 (zymogen granule membrane) (*GP2, ZAP75*); potassium voltage-gated channel, subfamily G, member 2 (*KCNG2*); cAMP-specific 3′,5′-cyclic phosphodiesterase 4D (*PDE4D*); killer cell immunoglobulin-like receptor, three domains, long cytoplasmic tail, 3 (*KIR3DL3*); inositol polyphosphate-5-phosphatase (*INPP5A*). We were also able to identify types of mutations in these genes. Namely, we identified nucleotide substitutions in *KIR3DL3*, *INPP5A* and *KCNG2*, multiple nucleotide substitutions in *SGCG*, *PDE4D* and *LHFPL3*, while *HTR4*, *ITGB1*, *CPS1*, *PROS1* and *GP2* were carrying both multiple substitutions and 2 nucleotide deletions (Fig. 3). We then analyzed eight out of 11 identified genes regarding the expression profile of these genes in glial cells as a basic criterion.

### Association of identified DNA alterations with genomic instability and clinicopathological parameters

Alterations of eight out of eleven identified genes were further examined in relation to the level of total, microsatellite and chromosomal genomic instability, tumor grade (grade III AA or grade IV GBM), age and sex (Tables 2 and 3). There was no statistical significance in correlation of identified DNA alterations with age and sex of the patients.

**Table 2:** Association between the frequency of altered genes, clinicopathological parameters and genomic instability.

		*LHFPL3*			*SGCG*			*PDE4D*			*HTR4*		
	Total cNP	NP	%	p	NP	%	p	NP	%	p	NP	%	p
Parameter													
Total	30	10	33.3		8	26.6		9	30		8	26.6	
**Glioma subtype**													
AA^a^	8	2	25.0	0.45	3	37.5	0.36	2	25.0	0.55	3	37.5	0.36
GBM	22	8	36.4		5	22.7		7	31.8		5	22.7	
**Sex**													
Male	19	8	42.1	0.18	5	26.3	0.64	5	26.3	0.43	5	26.3	0.64
Female	11	2	18.2		3	27.3		4	36.4		3	27.3	
**Age**													
≥50y	24	10	41.7	0.06	5	20.8	0.17	7	29.2	0.42	5	20.8	0.17
<50y	6	0	00.0		3	50.0		2	33.3		3	50.0	
**Genomic instability total**													
Low	13	1	7.7	**0.005** b	5	38.5	0.19	3	23.1	0.38	5	38.5	0.19
High	17	9	52.9		3	17.6		6	35.3		3	17.6	
**Microsatellite instability**													
Low	14	4	28.6	0.45	3	21.4	0.42	6	42.8	0.15	3	21.4	0.42
High	16	6	37.5		5	31.2		3	18.8		5	31.2	
**Chromosomal instability**													
Low	13	2	15.4	0.07	7	53.8	**0.005**	1	7.7	**0.02**	7	53.8	**0.005**
High	17	8	47.1		1	5.9		8	47.1		1	5.9	

^a^ AA, anaplastic astrocytoma; GBM, glioblastoma multiforme;

^b^ Bold indicates statistically significant values;

*LHFPL3*- lipoma HMGIC fusion partner-like 3; *SGCG*- sarcoglycan, gamma; *PDE4D*- cAMP-specific 3′,5′-cyclic phosphodiesterase 4D; *HTR4*-5-hydroxytryptamine (serotonin) receptor 4.^c^ NP, number of patients per group.;

**Table 3:** Association between the frequency of altered genes, clinicopathological parameters and genomic instability.

		*ITGB1*			*INPP5A*			*CPS1*			*PROS1*		
	Total cNP	NP	%	p	NP	%	p	NP	%	p	NP	%	p
Parameter													
Total	30	8	26.6		13	43.3		8	26.6		8	26.6	
**Glioma subtype**													
AA^a^	8	3	37.5	0.36	5	62.5	0.19	3	37.5	0.36	3	37.5	0.36
GBM	22	5	22.7		8	36.4		5	22.7		5	22.7	
**Sex**													
Male	19	5	26.3	0.64	10	52.6	0.17	5	26.3	0.64	5	26.3	0.64
Female	11	3	27.3		3	27.3		3	27.3		3	27.3	
**Age**													
≥50y	24	5	20.8	0.17	12	50.0	0.16	5	20.8	0.17	5	20.8	0.17
<50y	6	3	50.0		1	16.7		3	50.0		3	50.0	
**Genomic instability total**													
Low	13	5	38.5	0.19	5	38.5	0.46	5	38.5	0.19	5	38.5	0.19
High	17	3	17.6		8	47.0		3	17.6		3	17.6	
**Microsatellite instability**													
Low	14	3	21.4	0.42	5	35.7	0.34	3	21.4	0.42	3	21.4	0.42
High	16	5	31.2		8	50.0		5	31.2		5	31.2	
**Chromosomal instability**													
Low	13	7	53.8	**0.005** b	4	30.8	0.20	7	53.8	**0.005**	7	53.8	**0.005**
High	17	1	5.9		9	52.9		1	5.9		1	5.9	

^a^ AA, anaplastic astrocytoma; GBM, glioblastoma multiforme;

^b^ Bold indicates statistically significant values;

*ITGB1*- integrin, beta 1; *INPP5A*- inositol polyphosphate-5-phosphatase; *CPS1* - carbamoyl-phosphate synthetase 1, mitochondrial; *PROS1* - protein S (alpha).^c^ NP, number of patients per group;

*LHFPL3* was altered in 10 out of 30 patients (33.3%), predominantly in grade IV glioblastoma samples (36.4% vs. 25% in grade III anaplastic astrocytoma). It was detected in significantly higher percentage in samples with high level of total genomic instability (52.9% vs. 7.7% of samples with low level of total instability, p = 0.005). The same trend was observed for microsatellite (37.5% of samples with high vs. 28.6% of samples with low level) and chromosomal instability (47.1% of samples with high vs. 15.4% of samples with low level), but without statistical significance.

Alterations of *SGCG*, *HTR4*, *ITGB1*, *CPS1* and *PROS1* were detected in 26.6% of samples (8 out of 30 patients) and were slightly increased in patients with anaplastic astrocytoma (37.5%) compared to GBM (22.7%), as well as in the samples with low level of total genomic instability (38.5% vs. 17.64% with high instability). Statistical significance was observed in case of chromosomal instability, were 53.8% of patients with low level of CIN had alterations in these genes compared to only 5.9% of patients with high level of CIN (p = 0.005).

*PDE4D* was changed in 9 out of 30 patients (30%) and distributed almost equally in grade III and grade IV glioma (25% vs. 31.8% respectively). Altered *PDE4D* is associated with high level of chromosomal instability (p = 0.02) because it was detected in 47% of patients with high level of CIN, compared to 7.7% of patients with low level of this type of instability (Table 2).

The most frequently present alteration was in *INPP5A* gene, detected in 43.3% of patients, predominantly in patients with anaplastic astrocytoma (62.5% vs. 36.4% of GBM patients), and patients with high level of genomic instability (Table 3).

### Correlation analysis of identified DNA alterations and *p53*, *PTEN*, *p16* and *EGFR* alterations

We focused on four most frequently altered genes (*p53*, *PTEN*, *p16* and *EGFR*) in the genetic pathways of primary and secondary glioma [3] and analyzed their alterations in our set of samples. Alterations of *p53* were detected in 12 samples (40%), preferentially anaplastic astocytoma (p = 0.03), as reported in our previous paper [15]. LOH analyses of *PTEN* revealed that all 30 examined tumor specimens were heterozygous for at least one of the examined loci and 66.7% (20/30) of them demonstrated LOH. *p16* was analyzed for the loss of heterozygosity and homozygous deletions, two most common mechanisms for the inactivation of this tumor suppressor. LOH was detected in 14 out of 30 patients (46.7%). Homozygous deletions were studied by combination of differential PCR and fragment analysis and were detected in 11 out of 30 patients (36.7%). Overall, alterations of *p16* were present in 18 samples (60%).

Amplification of *EGFR* gene, assessed by differential PCR, was detected in 13 samples (43.3%). The Fisher exact test revealed that the frequency of *PTEN* and *EGFR* alterations was significantly higher in higher grade tumors (GBM) in comparison to anaplastic astrocytoma (82% vs 25%, p = 0.007 for *PTEN* and 54.5% vs. 12.5%, p = 0.047 for *EGFR*). Alterations of p16 were almost equally present in both histological subtypes (data not shown).

Next, we correlated obtained results with the presence of newly identified DNA alterations from AP-PCR DNA profiles of all patients (Tables 4 and 5). Remarkably, alterations of *PDE4D* were more frequently present in patients with wild-type *p53* and *p16* (p = 0.04 and p = 0.05, respectively). On the other hand, altered *INPP5A* was significantly associated with wild-type *EGFR* (p = 0.05). Interestingly, there was no significant correlation between loss of *PTEN* and any of the identified genes.

**Table 4:** Association of the frequency of identified genes with *p53*, *PTEN*, *p16* and *EGFR* alterations.

		*LHFPL3*			*SGCG*			*PDE4D*			*HTR4*		
	Total bNP	NP	%	p	NP	%	p	NP	%	p	NP	%	p
Parameter													
***p53***													
YES	12	5	41.7	0.34	4	33.3	0.40	1	8.3	**0.04** a	4	33.3	0.40
NO	18	5	27.8		4	22.2		8	44.4		4	22.2	
***PTEN***													
YES	20	7	35.0	0.56	4	20.0	0.23	6	30.0	0.67	4	20.0	0.23
NO	10	3	30.0		4	40.0		3	30.0		4	40.0	
***p16***													
YES	18	5	27.8	0.34	6	33.3	0.28	3	16.7	**0.05**	6	33.3	0.28
NO	12	5	41.7		2	16.7		6	50.0		2	16.7	
***EGFR***													
YES	13	5	38.4	0.45	5	27.8	0.19	3	23.1	0.38	5	27.8	0.19
NO	17	5	29.4		3	25.0		6	35.3		3	25.0	

^a^ Bold indicates statistically significant values;

*LHFPL3*- lipoma HMGIC fusion partner-like 3; *SGCG*- sarcoglycan, gamma; *PDE4D*- cAMP-specific 3′,5′-cyclic phosphodiesterase 4D; *HTR4*-5-hydroxytryptamine (serotonin) receptor 4.^b^ NP, number of patients per group;

**Table 5:** Association of the frequency of identified genes with *p53*, *PTEN*, *p16* and *EGFR* alterations.

		*ITGB1*			*INPP5A*			*CPS1*			*PROS1*		
	Total bNP	NP	%	p	NP	%	p	NP	%	p	NP	%	p
Parameter													
***p53***													
YES	12	4	33.3	0.40	7	58.3	0.16	4	33.3	0.40	4	33.3	0.40
NO	18	4	22.2		6	33.3		4	22.2		4	22.2	
***PTEN***													
YES	20	4	20.0	0.23	7	35.0	0.18	4	20.0	0.23	4	20.0	0.23
NO	10	4	40.0		6	60.0		4	40.0		4	40.0	
***p16***													
YES	18	6	33.3	0.28	8	44.4	0.59	6	33.3	0.28	6	33.3	0.28
NO	12	2	16.7		5	41.7		2	16.7		2	16.7	
***EGFR***													
YES	13	5	27.8	0.19	3	23.1	**0.05** a	5	27.8	0.19	5	27.8	0.19
NO	17	3	25.0		10	58.8		3	25.0		3	25.0	

^a^ Bold indicates statistically significant values;

*ITGB1*- integrin, beta 1; *INPP5A*- inositol polyphosphate-5-phosphatase; *CPS1* - carbamoyl-phosphate synthetase 1, mitochondrial; *PROS1* - protein S (alpha).^b^ NP, number of patients per group;

### Identified DNA alterations in relation to follow-up

**Figure 4:**
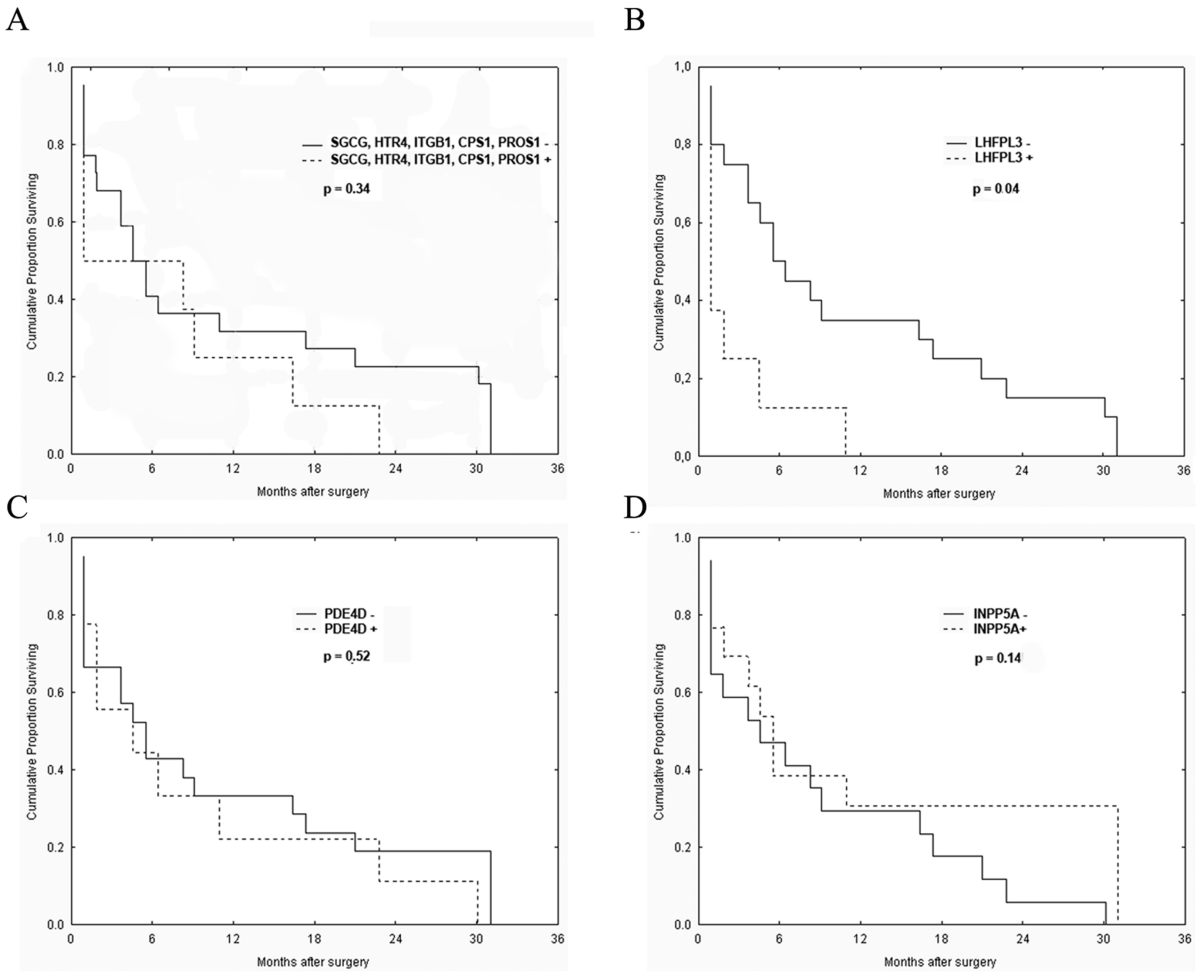
Kaplan–Meier survival curves. (**A**) Patients without alterations in *SGCG, HTR4, ITGB1, CPS1* and *PROS1* had tendency for better survival; (**B**) patients with alterations in *LHFPL3* lived significantly shorter (p = 0.04); (**C**) alterations of *PDE4D* had no impact on patients' survival (**D**) alterations of *INPP5A* had no impact on patients' survival.

The overall survival of 30 patients was evaluated in relation to presence or absence of alterations of newly detected genes. In all cases patients were divided into two groups, those with and those without altered gene. Although there was no statistically significant difference among patients' survival rate, it was noticed that patients with alterations of *SGCG*, *HTR4*, *ITGB1*, *CPS1* and *PROS1* had shorter survival rate (Fig. 4A). The survival of patients with alterations of *LHFPL3* was significantly shorter than the survival of patients without these alterations (p = 0.04, Fig. 4B), while alterations of *PDE4D* and *INPP5A* seem to have no impact on patients' survival (Fig. 4C,D).

## Discussion

The purpose of this study was to identify altered genes in 30 human glioma samples specifically associated with progression and outcome of this type of tumor. Our study revealed that AP-PCR fingerprinting is very useful in the identification and characterization of the regions of the genome of human glial tumors that have undergone alterations. The high level of genomic instability (median 34%) observed in all grade III and grade IV tumor samples analyzed in this study indicates the possibility of involvement of many more genes than those currently known to be of importance for development of these tumors. Our results indicate that alterations of eight out of eleven identified genes might have an important role in pathogenesis of glial tumors, while detected changes in *GP2*, *KCNG2* and *KIR3DL3* should be considered as passenger mutations, since these genes are not expressed in glial cells. We assume that alterations of these three genes occur during clonal expansion of the genetically unstable tumor cells, and represent a consequence of detected significant level of genomic instability. [15]. Genetic alterations identified in our study are infrequent in TCGA database samples (http://tcga-portal.nci.nih.gov), and represent an addition to other multi-institutional studies reports.

Alterations of lipoma *HMGIC* fusion partner (*LHFPL3*) were more frequently detected in grade IV GBM, as well as in older patients and samples with high level of genomic instability which was shown to be present in *de novo* glial tumors [15]. This gene, located at the long arm of chromosome 13, acts as a translocation partner of *HMGIC* in lipoma with t(12;13) [26] and the latest work of Nagaishi et al. [27] showed association of its amplifications with mesenchymal differentiation in gliosarcoma. According to our results, multiple nucleotide substitutions detected in this gene could also have impact on glioma invasiveness, especially considering significantly shortened survival rate of the patients carrying these alterations.

On the other hand, alterations of *SGCG*, *HTR4*, *ITGB1*, *CPS1*, *PROS1* and *INPP5A* were detected predominantly in anaplastic astrocytoma, while alterations of *PDE4D* were present in both glioblastoma subtypes. Most importantly, the majority of identified genes have a significant role in signal transduction or cell adhesion, the important processes for cancer development and progression, but were not previously associated with glioma pathogenesis. Namely, *HTR4*, and *PDE4A* are among key players in cAMP signaling [28], [29], while *INPP5A* regulates the level of another two secondary messengers, phosphatidylinositol (1,4,5)-trisphosphate and inositol-1,3,4,5-tetrakisphosphate [30].

Inverse relation between cAMP levels and the degree of malignancy was already shown in several types of brain tumors [31], and overexpression of *PDE4* was considered to be one of the main mechanisms for reduction of its intracellular level [32]. In our study, alterations of *PDE4D* were more frequently found in patients carrying wild-type *p53* and *p16* genes, indirectly suggesting that decrease of cAMP in glial tumors might be one of the mechanisms for regulation of the activity of these tumor suppressors.

Serotonin (5-hydroxytryptamine; 5HT) and its receptors also regulate cAMP production [33]. Alterations of *HTR4* gene, frequently present in our set of samples, were not previously detected in glioma. On the other hand, there are several reports on different tumor types (breast cancer, small cell lung cancer, prostate cancer and adenocortical adenoma) indicating that tumor cells acquire alterations in the 5-HT signaling that favor tumor-promoting actions and have a fundamental role in tumor growth, differentiation and gene expression [34], [35].

As already mentioned, InsP3 5-phosphatase (INPP5A) functions mostly as a signal-terminating enzyme with implication for several cellular processes, including proliferation [36]. It was shown that loss of INPP5A was an early event in development of cutaneous squamous cell carcinoma [37]. Speed et al. [38] showed that absence or loss of this enzyme activity was also associated with transformation of NRK cells, while Mengubas et al. [39] connected decrease of inositol polyphosphate 5-phosphatase activity with several human leukaemias.

*INPP5A* is located in the short arm of chromosome 10 (10q26.3), and LOH of this region represent the most frequent genetic abnormality in both primary and secondary GBM, due to the presence of multiple tumor suppressor genes in this chromosomal region [3], [40].

Our study also showed that alterations of *INPP5A* were significantly more frequent in samples without *EGFR* amplifications. This is in accordance with finding that chronic elevation of inositol phosphate in unstimulated human fibroblast cells leads to increase in basal calcium level and mitogenesis independently of growth factors [38].

Next, our results suggest that CPS1, one of the five key enzymes of the urea cycle [41] might also be aberrant in gliomas. Numerous point mutations and polymorphisms in this gene diversely affecting its biological function have been previously identified [42]. Altered expression of *CPS1* was demonstrated in gastric cancer [43], hepatocellular carcinoma [44] and small-intestinal adenocarcinoma [45], but there are no data on glial tumors published so far. In our set of samples alterations of *CPS1* were mainly found in grade III anaplastic astrocytoma with low level of genomic instability, suggesting potential role of this enzyme in neoplastic progression of secondary glioblastoma.

Protein products of *SGCG* and *PROS1* are glycoproteins involved in cell adhesion [46], [47], while transmembrane receptor ITGB1 has additional important role in cell signaling and regulation of cell cycle [48]. Data about their role in cancer progression are scarce, consisting of a few reports on different types of cancer. Downregulation of SGCG was detected in NSCLC [49] and breast cancer [50], while Saraon et al. [51] revealed elevation of PROS1 in high grade aggressive prostate cancer. Contrary to this, PROS1 seems to be downregulated in anaplastic meningiomas [52]. To the best of our knowledge, this is the first study that shows association between alterations of these two genes and glioma progression.

Integrin family members, due to their biological features, have numerous functions in cancer progression, especially in invasion and metastasis formation. The action of integrins has been studied in malignant melanoma, breast, prostate and pancreatic cancer [53], [54], [55], [56]. There are also reports suggesting important roles of ITGB1 in glioma invasiveness and resistance to temozolomide treatment [57], which are in accordance with our findings.

Our results indicate that alterations of *SGCG*, *PROS1* and *ITGB1* genes appeared prevalently in grade III astrocytomas and resulted in shorter survival of patients, implying more aggressive nature of tumors with this genetic signature.

In conclusion, our study revealed that AP-PCR fingerprinting was extremely useful in the identification and characterization of the regions of glial tumor genome that have undergone alterations in comparison to their corresponding controls. Most importantly, we were able to identify genes associated with glioma pathogenesis, previously not related to this type of cancer. In particular, majority of identified genes play an important role in cell signaling, regulation of cell cycle, and cell adhesion, therefore participating in processes severely affected during malignant transformation. Further investigations of the detected genes on larger sample size, as well as *in vitro* studies, are under way, with special emphasize on clarifying the mechanisms of their action in progression of malignant glioma.
